# Old tech but not low tech: telephone-based treatment provision for substance use

**DOI:** 10.3389/fpsyt.2024.1351816

**Published:** 2024-03-19

**Authors:** Mary R. Walton, Augustine W. Kang, Courtney DelaCuesta, Ariel Hoadley, Rosemarie Martin

**Affiliations:** ^1^ CODAC Behavioral Healthcare, Cranston, RI, United States; ^2^ Rosemarie Martin Laboratory, Department of Behavioral and Social Sciences, School of Public Health, Brown University, Providence, RI, United States

**Keywords:** substance use disorder treatment, opioid treatment, telemedicine, audio-only, telephone, hybrid approach

## Abstract

The future of telemedicine for substance use treatment hangs by a thread, as the United States awaits approval of proposed regulations and laws to increase care access in light of the 2022 Centers for Medicare and Medicaid Services revisions allowing for audio-only care. Telemedicine improves patient care access and outcomes. Audio-only telemedicine can be an effective and viable modality for individuals without technology resources (devices, internet services, and literacy), those with reduced telehealth service utilization (Black individuals or those with unstable housing, who are older, with low income, or with low education), and those living in rural locations. Studies suggest that telephone visits for buprenorphine treatment are well-accepted by patients and providers, making telephone visits essential in care access to reduce disparities. Telephone counseling for patients in substance use treatment is convenient, flexible, and empowering and can augment therapeutic alliances and treatment goals. Both providers and patients advocate for patient-centered hybrid care to include telephone-only treatment, which enhances service productivity and care access; reduces no-show rates, costs, and stigma; and is sustainable. Numerous solutions can expand technology access, proficiency, assimilation, and trust. Despite being “old” technology, the telephone remains an essential resource for substance use treatment.

## Introduction

The future of telemedicine for substance use disorder (SUD) treatment is at a crossroads, as 2023 heralded proposed regulations, law changes, and service revisions that seek to increase care access. Some of these changes amend the Ryan Haight Act to authorize schedule III or IV controlled substances to be prescribed without initial in-person evaluation:

▪ The General Telemedicine Rule: Proposed Drug Enforcement Administration (DEA) regulation allowing telemedicine prescribing of *non-narcotic* schedule III–V controlled substances without a prior in-person medical evaluation; evaluation can include audio-only technology when the patient cannot or will not use audio-video technology (1); extended until December 31, 2024.
▪ The Buprenorphine Rule: Proposed regulation amendment by the DEA and the Department of Health and Human Services to expand telemedicine maintenance or withdrawal management prescribing of schedule III–V *narcotic* drugs (buprenorphine) to include audio-only telemedicine (2).
▪ Pending Telehealth Response for E-prescribing Addiction Therapy Services (TREATS) Act: Permanently expands SUD treatment by permitting induction of buprenorphine treatment via audio-visual telehealth (3); reintroduced on November 1, 2023.
▪ Pending Creating Opportunities Now for Necessary and Effective Care Technologies for Health (CONNECT 2023) Act of 2023: Primarily proposes to expand coverage of telehealth services under Medicare, such as types of technology that may be used, removing originating geographic sites, allowing more eligible healthcare professionals to use telehealth, and removing in-person requirements for some services (4); reintroduced June 15, 2023.
▪ US Centers for Medicare and Medicaid Services permitted in 2022 audio-video communications technology to include initiating buprenorphine treatment or *audio-only technology when audio-video technology is unavailable* and continuing audio-only therapy, counseling, and periodic assessments when the patient cannot or will not use audio-video technology (5).

Two days before the COVID-19 State of Emergency ended in May 2023, the DEA and the Substance Abuse and Mental Health Services Administration (SAMHSA) issued a temporary extension of COVID telemedicine flexibilities for the General Telemedicine and Buprenorphine Rules due to needing further review of the 38,000+ public comments (6). The DEA held two listening sessions in September 2023 for the General Telemedicine Rule to receive feedback on telemedicine practices and protections for detecting and preventing controlled substance diversion if passed. Making telemedicine a permanent fixture to include audio-only services can increase entry to care in the opioid epidemic and address persistent COVID-19 transmission and a lack of technology access among populations at risk for health disparities who comprise a large portion of individuals with SUDs.

## Discussion

Before COVID-19 in 2019, SUD services using telemedicine modalities were only at 17% (7). Increasing the uptake of telemedicine modalities in SUD treatment is critical due to documented improvements in patient care access and outcomes (8, 9), adherence (10), and decreased transportation time and travel anxiety and costs (11, 12). Telemedicine used in SUD had comparable efficacy with in-person care in substance use reduction, retention in treatment services, and treatment satisfaction (8, 10, 11).

Telemedicine is comprised of two primary modalities: audio-visual and audio-only platforms. Video platforms have clear benefits over telephone platforms, enabling the ability to visualize the patient and observe non-verbal cues. However, fewer patients have access to video telemedicine and are often also at risk for health disparities. Those in opioid treatment with limited income and resources found video visits unfeasible (9). Patients want choices and accept both telemedicine modalities (13).

### Audio-only telemedicine

Audio-only telemedicine in SUD can be an effective and viable telemedicine modality for those with less technology resources (devices, internet services, and literacy) (9) or less likely to utilize video telehealth services (Black individuals or those with unstable housing (8, 9), who are older, with low income, or with low education (9, 11)) and in rural locations. Moreover, studies have demonstrated patients’ ability to utilize telephones for SUD treatment, with telephone services providing greater patient accessibility, affordability, and sustainability than video technologies (9, 14). An overlap exists among populations experiencing homelessness and those with SUD. In addition, 94% of those experiencing homelessness own a mobile phone (15), suggesting opportunities to increase audio-only telemedicine for SUD care in underserved populations.

Audio-only telemedicine medical visits for buprenorphine treatment can bridge opioid treatment access to diminish the technological divide in healthcare disparities (8). An early COVID survey (16) demonstrated that almost half (48%) of buprenorphine providers performed buprenorphine inductions via phone. Buprenorphine providers using telemedicine for medical visits report reduced no-show rates and improved patient interaction quality, engagement (9), and compliance (17). Frost et al. in a large (n = 17,182) Veterans Health Administration cross-sectional study (8) during the COVID-19 pandemic reported significantly higher retention among telemedicine visits for buprenorphine compared to in-person-only visits for both new and continuing patients. Black individuals and people experiencing homelessness or unstable housing were more likely to access buprenorphine through telephone visits only. Another prospective study (9) reported no significant difference in retention 30 days after telephone *vs.* video initiation of buprenorphine treatment, suggesting that audio-only does not necessarily result in lower-quality care. Similarly, a longitudinal study (18) in low-threshold care of 150 patients demonstrated an 80% retention rate after 1 year in audio-only buprenorphine treatment, primarily in Black men. These studies suggest that telephone visits for buprenorphine treatment are well-accepted by patients and providers and that telephone visits are essential in increasing care access to reduce disparities, highlighting the importance of passing the proposed DEA regulations for SUD care, particularly buprenorphine.

Age is a notable consideration in using telehealth as a modality for SUD treatment. In a 2022 study (9) on over 4,500 buprenorphine treatment encounters in almost 800 patient records, those older than 50 were significantly more likely to use audio telehealth as a principal visit mode. In patients less likely to use telephones as a main visit modality, younger age (<50 years) was not found to be directly significant; however, this group reported higher rates of having overdosed in the previous 6 months, which may be ascribed to younger age. Frost et al. (8) demonstrated that patients at least 65 years old had more frequent telehealth visits and were less likely to engage in video visits. Those with more frequent video visits were aged 30 to 44 years. Limitations of both studies were a lack of racial diversity, reporting a vast majority of White participants. As the Baby Boomer generation increases in longevity, SUDs among older persons are an escalating health concern. The prevalence of adults aged 65 years and older who meet SUD criteria is over 4 million—a fourfold increase from approximately 1 million in 2018, affecting both genders and all racial/ethnic groups (19). This surge results in higher numbers and proportion of SUDs than previous older populations, necessitating greater treatment demand (20). The telephone is hence a crucial component in maintaining SUD care, given the propensity for audio telehealth in older populations facing retirement and medical comorbidities. Randomized controlled trials are needed to better understand demographic roles, such as race and age, in hybrid approaches to SUD care.

### Audio-only substance use counseling

Therapeutic alliances are critical for treatment engagement and retention, can reduce treatment drop-out rates, and can increase the scope of patient goals and tasks in addiction treatment. A meta-analysis (21) of 295 studies reported the therapeutic alliance to be the strongest factor in positive treatment outcomes in psychotherapy, with the SUD therapeutic alliance having only a slightly lower effect size. In SUD treatment, stronger therapeutic alliances led to a greater decline in distress during treatment (22). Positive telemedicine experiences in SUD counseling, therapeutic alliance, and attendance are reported as well (10). Moreover, telephone-administered psychotherapy improved depression symptoms to a similar extent to in-person treatment modalities per a systematic review and meta-analysis (23).

### Substance use disorder telephone counseling experiences

A cross-sectional survey (12) of 237 patients conducted as part of a risk management project at a multi-site opioid treatment program during the COVID pandemic reported positive SUD telephone counseling experiences by patients, such as convenience, flexibility, time- and cost-effectiveness, empowerment in substance use management, and reduction in transportation barriers and time-related family conflicts. Patients felt supported by their counselors and experienced increased access to counselors and frequency of sessions, with a higher percentage reporting adequate privacy for sessions than inadequate privacy; this resulted in a better therapeutic alliance and greater patient accountability to the counselor. Overall, satisfaction with phone counseling was high for patients (81%) and counselors (69%), with only 2% reporting any form of dissatisfaction.

Patients more comfortable with phone *vs.* face-to-face counseling sessions were 8.3 times as likely to experience relationship improvements with counselors in the virtual setting and were almost five times as likely to feel that phone counseling sessions were more helpful for addressing substance use. The patients who self-reported depression and felt more comfortable with phone counseling sessions were three times as likely to feel that phone sessions were more helpful for their depression and seven times as likely to feel phone counseling supported their recovery. Patients with anxiety who were more comfortable with phone counseling were eight times as likely to experience patient–counselor relationship improvements virtually, 5.4 times as likely to feel phone counseling was more helpful for addressing substance use, and 7.8 times as likely to support recovery. Approximately 93% of samples had adequate privacy during counseling sessions, despite initial concerns surrounding patient phone access, ample data coverage, or sufficient privacy for remote counseling sessions. The comfort level with telephone counseling reported in this study is the same or greater compared with pre-COVID research examining telephone counseling modality for SUDs (24).

### Hybrid approaches to substance use counseling

Individualized treatment approaches must be employed to decrease the digital divide and disparities in SUD treatment to improve care outcomes, such as initiating medications, assessments, and referrals, as well as tailor counseling and other care approaches. Both providers and patients demonstrate a preference for hybrid care models (13), a combination of in-person and virtual approaches. Hybrid care is patient-centered, enhances service productivity (13), continues social distancing, reduces no-show rates (17) and costs for facilities (13) and patients, and eliminates the stigma associated with being seen attending services (17). Hybrid medical and counseling telemedicine visits are more accommodating, convenient, and time- and cost-effective and reduce transportation needs and other barriers to care, enabling care access and retention. Espousal of hybrid telemedicine use for all virtual platforms in medication for opioid use disorder treatment is prudent in keeping patients in care.

Audio-only telemedicine can be an effective platform in a hybrid approach to SUD treatment, particularly for marginalized persons lacking resources and older populations with accumulating comorbidities. Addressing mental health issues and substance use through audio-telemedicine can also improve the therapeutic alliance and recovery support, requiring the need to augment access to technology and existing SUD treatment. Some solutions to expanding technology access include increasing geographical care catchment ranges (13); library loans; increasing affordability of telephones, internet devices, and services; technology training for staff and patients to increase proficiency, trust, and assimilation; and facilities providing private video or telephone care access for patient intakes, assessments, medical visits, counseling, and referrals in areas such as emergency departments, syringe exchange programs, safe injection sites, and mobile and rural clinics. Future studies to inform policy could investigate how hybrid in-person and virtual approaches enhance the patient treatment experience and address sociodemographic disparities in efforts to decrease substance use and increase recovery stability. The old-tech but not low-tech telephone is a critical resource and must find permanence in SUD treatment.

## Data availability statement

The raw data supporting the conclusions of this article will be made available by the authors, without undue reservation.

## Author contributions

MW: Conceptualization, Investigation, Project administration, Writing – original draft, Writing – review & editing. AK: Data curation, Formal Analysis, Methodology, Validation, Writing – review & editing. CD: Conceptualization, Data curation, Formal Analysis, Investigation, Methodology, Writing – review & editing. AH: Conceptualization, Writing – review & editing, Formal Analysis. RM: Conceptualization, Investigation, Project administration, Resources, Supervision, Writing – review & editing.

## References

[1] Drug Enforcement Administration, Department of Justice. Telemedicine prescribing of controlled substances when the practitioner and the patient have not had a prior in-person medical evaluation. Fed Regist. (2023) 88:12875–12890.

[2] Drug Enforcement Administration. Expansion of induction of buprenorphine via telemedicine encounter. Fed Regist. (2023) 88:12890–12906.

[3] Telehealth Response for E-prescribing Addiction Therapy Services Act (TREATS). S3.40, 117th Cong (2021). Available online at: https://www.congress.gov/bill/117th-congress/senate-bill/340/text (Accessed November 27, 2023).

[4] Creating Opportunities Now for Necessary and Effective Care Technologies (CONNECT) for Health Act of 2021, HR 2903, 117th Cong (2021-2022). Available online at: https://www.congress.gov/bill/117th-congress/house-bill/2903 (Accessed November 27, 2023).

[5] CMS.gov. Opioid treatment programs (OTPs) (2022). Available online at: https://www.cms.gov/medicare/medicare-fee-for-service-payment/opioid-treatment-program (Accessed January 29, 2023).

[6] Drug Enforcement dministration Department of Justice Substance Abuse and Mental Health Services Administration Department of Health Human Services. Temporary extension of COVID-19 telemedicine flexibilities for prescription of controlled medications. Fed Regist. (2023) 88:33037–43.

[7] Uscher-Pines L Cantor J Huskamp HA Mehrotra A Busch A Barnett M. Adoption of telemedicine services by substance abuse treatment facilities in the U.S. J Subst Abuse Treat. (2020) 117:108060. doi: 10.1016/j.jsat.2020.108060 32811631 PMC7308749

[8] Frost MC Zhang L Kim HM Lin LA. Use of and retention on video, telephone, and in-person buprenorphine treatment for opioid use disorder during the COVID-19 pandemic. JAMA Netw Open. (2022) 5:e2236298. doi: 10.1001/jamanetworkopen.2022.36298 36223118 PMC9557869

[9] Chang JE Lindenfeld Z Thomas T Waldman J Griffin J. Patient characteristics associated with phone versus video telemedicine visits for substance use treatment during COVID-19. J Addict Med. (2022) 16:659–65. doi: 10.1097/ADM.0000000000000985 PMC9653061 35195540

[10] Mark TL Treiman K Padwa H Henretty K Tzeng J Gilbert M. Addiction treatment and telehealth: review of efficacy and provider insights during the COVID-19 pandemic. Psychiatr Serv. (2022) 73:484–91. doi: 10.1176/appi.ps.202100088 34644125

[11] Pham H Lin C Zhu Y Clingan SE Lin L Mooney LJ. Telemedicine-delivered treatment for substance use disorder: A scoping review. J Telemed Telecare. (2023) 0(0):1357633X231190945. doi: 10.1177/1357633X231190945 PMC11444076 37537907

[12] Martin R Kang AW DeBritz AA Walton MR Hoadley A DelaCuesta C. Medication for opioid use disorder service provision and telephone counseling: a concurrent mixed-methods approach. Int J Environ Res Public Health. (2021) 18:6163. doi: 10.3390/ijerph18116163 34200312 PMC8201197

[13] Thomas EE Taylor ML Ward EC Hwang R Cook R Ross J. Beyond forced telehealth adoption: A framework to sustain telehealth among allied health services. J Telemed Telecare. (2022) 0(0):1357633X221074499. doi: 10.1177/1357633x221074499 PMC10928953 35130099

[14] Molfenter T Roget N Chaple M Behlman S Cody O Hartzler B. Use of telehealth in substance use disorder services during and after COVID-19: online survey study. JMIR Ment Health. (2021) 8:e25835. doi: 10.2196/25835 33481760 PMC7895293

[15] Rhoades H Wenzel S Rice E Winetrobe H Henwood B. No digital divide? Technology use among homeless adults. J Soc Distress Homeless. (2017) 26:73–7. doi: 10.1080/10530789.2017.1305140 PMC6516785 31097900

[16] American Academy of Addiction Psychiatry. First glance: COVID-19 buprenorphine provider survey report (2020). Available online at: https://www.aaap.org/first-glance-covid-19-buprenorphineprovider-surveyreport/ (Accessed November 27, 2023).

[17] Edinoff AN Kaufman SE Chauncy TM Erwin AP Russo KM Nelson ME. Addiction and COVID: Issues, challenges, and new telehealth approaches. Psychiatry Int. (2022) 3:169–80. doi: 10.3390/psychiatryint3020014

[18] Harris R Rosecrans A Zoltick M Willman C Saxton R Cotterell M. Utilizing telemedicine during COVID-19 pandemic for a low-threshold, street-based buprenorphine program. Drug Alcohol Depend. (2022) 230:109187. doi: 10.1016/j.drugalcdep.2021.109187 34890927 PMC8619879

[19] Substance Abuse and Mental Health Services Administration. Key substance use and mental health indicators in the United States: Results from the 2021 National Survey on Drug Use and Health (2022). Available online at: https://www.samhsa.gov/data/report/2021-nsduh-annual-national-report (Accessed February 27, 2024).

[20] Yarnell S Li L MacGrory B Trevisan L Kirwin P. Substance use disorders in later life: A review and synthesis of the literature of an emerging public health concern. Am J Geriatr Psychiatry. (2020) 28:226–36. doi: 10.1016/j.jagp.2019.06.005 31340887

[21] Flückiger C Del Re AC Wampold BE Horvath AO. The alliance in adult psychotherapy: A meta-analytic synthesis. Psychother (Chic). (2018) 55:316–40. doi: 10.1037/pst0000172 29792475

[22] Urbanoski KA Kelly JF Hoeppner BB Slaymaker V. The role of therapeutic alliance in substance use disorder treatment for young adults. J Subst Abuse Treat. (2012) 43:344–51. doi: 10.1016/j.jsat.2011.12.013 PMC3345301 22285833

[23] Castro A Gili M Ricci-Cabello I Roca M Gilbody S Perez-Ara MA. Effectiveness and adherence of telephone-administered psychotherapy for depression: A systematic review and meta-analysis. J Affect Disord. (2020) 260:514–26. doi: 10.1016/j.jad.2019.09.023 31539688

[24] Steinkamp JM Goldblatt N Borodovsky JT LaVertu A Kronish IM Marsch LA. Technological interventions for medication adherence in adult mental health and substance use disorders: A systematic review. JMIR Ment Health. (2019) 6:e12493. doi: 10.2196/12493 30860493 PMC6434404

